# Hydrastis Canadensis in the Treatment of Bronchial Catarrh

**Published:** 1897-06

**Authors:** 


### HYDRASTIS CANADENSIS IN THE TREATMENT OF BRONCHIAL CATARRH.

In the *Centralblatt fur Innere Medicine* for May 1st, Dr. M. Saenger, of Magdeburg, gives his impressions of hydrastis as a remedy for bronchial catarrh. It seems that some six years ago he prescribed it for a patient whom he was treating for a tuberculous affection of the larynx and apex catarrh, the immediate occasion of its employment being a trifling hæmoptysis caused by the patient lifting a heavy weight. Four days later Dr. Saenger saw the patient again, and learned from him that for three days there had been no blood in the expectoration, and, furthermore, that a tormenting cough with which he had suffered had completely disappeared, the expectoration had become decidedly less, and the character of the sputa had changed in that they were far less frequently greenish and tenacious than before. The patient, a man of intelligence, imputed all his improvement to the use of the medicine that had been ordered for him.

Although Dr. Saenger himself was properly skeptical as to this point, he tried hydrastis on another phthisical patient, not for the purpose of checking hæmoptysis, but to mitigate a troublesome cough with great difficulty of expectoration. In this case, too, there was great improvement. For the most part, the sputa lost their purulent admixture and became thinner. The patient declared that the medicine had given him more relief than he had obtained from the morphine, codeine, Dover's powder, apomorphine, and other like drugs that had previously been ordered for his cough. His night's rest was no longer disturged by coughing, he could breathe easier and deeper, he felt stronger, and he was better able to attend to his business, As in the first case mentioned, physical examination of the thorax showed a notable diminution of the bronchial catarrh.

Subsequently Dr. Saenger used hydrastis in a great number of cases of bronchitis, including those not dependent on tuberculous trouble. He found that in the initial stage of acute bronchial catarrh it was quite ineffectual, but that in the subsequent course of the disease it was beneficial, especially if the course was protracted and the sputa had lost their purely mucous character and assumed a muco-purulent aspect. He found the remedy particularly efficacious in chronic bronchitis, for it mitigated the cough strikingly, facilitated expectoration, changed the muco-purulent character of the sputa to a more mucous one, and decidedly diminished the physical signs.

As compared with opium and its derivatives, says Dr. Saenger, if hydrastis is not quite so prompt in its action in checking the cough, it is more enduring and its final effect is greater, for it acts upon the cause of the cough, producing a more or less complete disappearance of the catarrh. As an expectorant, it is at least equal to the other expectorants and solvents that are in use. So far as can be judged from physical exploration of the chest and from examination of the sputa, it far excels the other anti-catarrhal drugs in use. He states that he could not do without hydrastis now in the treatment of bronchial catarrh, acute as well as chronic, for it enables him to dispense with the use of opium and its derivatives almost entirely in the treatment of tuberculous subjects.

He has employed it in the form of the fluid extract almost exclusively. To adults he gives twenty, twenty-five or thirty drops four times a day, in a little sweetened water. In case it does not produce the expected effects, larger doses may be used. He has not found hydrastinine so trustworthy as the fluid extract. He has never observed dangerous or unpleasant effects from the doses of the fluid extract mentioned, but he remarks that very large doses may give rise to angina pectoris in the subjects of heart disease and in very debilitated persons.—*New York Medical Journal.*

